# Research on the Hydration Properties of C_4_A_3_S-CSH_2_ Cement System at Different Temperatures

**DOI:** 10.3390/ma13184000

**Published:** 2020-09-09

**Authors:** Min Li, Mingzhang Lan, Zhifeng Chen, Jianfeng Wang, Suping Cui, Yali Wang

**Affiliations:** 1 Faculty of Materials and Manufacturing, Beijing University of Technology, Beijing 100124, China; limiiyi@126.com (M.L.); lanmingzhang@bjut.edu.cn (M.L.); cuisuping@bjut.edu.cn (S.C.); wangyali1978@bjut.edu.cn (Y.W.); 2 Tangshan Polar Bear Building Materials Co. and Ltd., Tangshan 063705, China; lxchenzf@tspolarbear.com

**Keywords:** low temperature, calcium sulfoaluminate, AFt, strength

## Abstract

Sulphoaluminate cement has the advantage of low-temperature application performance, but its hydration mechanism at low temperatures is not yet clear. Anhydrous calcium sulfoaluminate (C_4_A_3_S) is the main mineral in the composition of sulfoaluminate cement clinkers. In this paper, C_4_A_3_S mixed with gypsum (CaSO_4_∙2H_2_O) to form a C_4_A_3_S-CSH_2_ cement system; X-ray diffraction (XRD), thermogravimetric analysis (TG-DTG), scanning electron microscopy (SEM) and mercury intrusion analysis (MIP) to clarify the effect of temperatures on the hydration properties of C_4_A_3_S-CSH_2_ cement system. The results showed that hydration of the C_4_A_3_S-CSH_2_ cement system could carry on at low temperatures, even at −15 °C. The main hydration product was ettringite. Low temperatures did not change the types of the hydration products, but the low temperature of 0 °C was more favorable for the formation of ettringite. The early hydration of the C_4_A_3_S-CSH_2_ cement system was inhibited by the decrease in temperature. However, hydration of the cement at 0 °C continued at a high rate after one day. Morphologies of the ettringite for the C_4_A_3_S-CSH_2_ cement system at −15 °C were needle-like structures, while they were of columnar structure at 0 °C. The compressive strength of samples at 0 °C reached 82 MPa, which is significantly higher than that at 20 °C.

## 1. Introduction

Calcium sulphoaluminate cements (CSA) are promoted as one of the most promising cementitious materials due to their outstanding properties such as lower CO_2_ emissions [1], fast setting and hardening, high early strength [2], impermeability [3] and good durability [4]. In recent years, CSA has been attracting more and more attention in the fast repairing of civil structures [5], waste stabilization/solidification [6], as expansion material for shrinkage compensation [7,8] and low-temperature construction projects [9]. C_4_A_3_S is the main mineral in the composition of sulphoaluminate cements clinkers. It has the advantages of high hydration activity, strong low temperature adaptability and high early strength. It is widely used in winter construction, emergency repairs, marine engineering, etc.

Traditionally, CSA was produced by mixing clinkers (ye’elimite, C_4_A_3_S) with different amounts of a calcium sulfate in combination with other phases, such as belite (C_2_S), lime and ferrite (C_4_AF), depending on the raw materials used [10]. Rapid reaction starts right after the contact of the dry blends with water and it releases significant amount of heat, which makes it possible to hydrate at low temperatures. The main resulting hydration products are ettringite (AFt) and monosulfate (AFm), together with amorphous aluminium hydroxide (AH_3_). Typically, the amount and reactivity of the added calcium sulfate determines the ettringite to monosulfate ratios. The hydration of C_4_A_3_S without calcium sulfate leads to the formation of AFm and amorphous AH_3_ as the dominant hydration products, however, the presence of calcium sulfate favors the formation of AFt [3,11,12].

Hydration properties of calcium sulfoaluminate cements are governed by a combination of factors [13,14,15,16,17,18,19], especially temperature. Previous studies showed that ambient temperature and hydration temperature play an important role in cement hydration kinetics, and changes in temperature will lead to variations in product types and distributions [16,17]. It was confirmed that increase in temperature could enhance the solubility of calcium sulfate and ettringite, resulting in the increase of sulfate ion concentration in pore solution. Moreover, metastable calcium aluminate hydrates such as CAH_10_, C_4_AH_13_ and C_2_AH_8_ tend to transform to C_3_AH_6_ at high temperatures. These transformations could lead to increase in porosity and a significant decrease in strength [19,20,21]. In addition, the hydration of sulphoaluminate cement at the early stage is very fast with huge and concentrated heat release, which makes it possible for concrete construction at low temperature. Tang [22] prepared 1.7 L of calcium sulphoaluminate cement paste at 20 °C to study the hydration properties of the C_4_A_3_S-CSH_2_ cement system at low temperatures. They reported that the internal temperature of the sulphoaluminate cement paste could increase by 5 °C within a day. Wang [23] concluded that low temperatures could not change the hydration products, but greatly impacted the amount and microstructures of the products. With the increasing interest in using sulphoaluminate cement as a candidate material for winter concrete construction projects, understanding of the hydration properties of the sulphoaluminate cements at low temperatures have become critical.

Past research and practice have shown that sulphoaluminate cement can be kept in the hydration reaction and reach the corresponding strength by relying on heat released by its own hydration as long as the sulphoaluminate cement is put into the mold at no less than 5 °C during winter construction. There are also many studies on the hydration reaction characteristics of sulfoaluminate cement under negative temperature curing, but there are few reports on the hydration process of sulfoaluminate cement under a completely negative temperature environments (raw materials, water, etc.).

In addition, when studying the low-temperature hydration of sulphoaluminate cement, the molding temperature is inconsistent with the curing environment temperature. Usually, the sulphoaluminate cement-based material is molded at 5 °C and then placed in a negative low temperature environment. Curing ignores the temperature difference between the initial temperature of the raw materials and the external environment. This interferes with the study of the macroscopic mechanical strength development law and microscopic hydration characteristics of sulphoaluminate cement under negative low temperature environments. Therefore, the task of studying the strength development law and hydration characteristics of sulphoaluminate cement under low temperature environments should be put on the agenda.

Therefore, this work uses the laboratory to prepare anhydrous calcium sulfoaluminate minerals and gypsum to form sulphoaluminate cement, to study the hydration characteristics of sulphoaluminate cement in low temperature environments, and to clarify the low-temperature hydration mechanism of C_4_A_3_S. At the same time, it further reveals the reason for the high later strength of cement under low temperature (0 °C) environments. This can not only provide effective technical means for determining the optimization and control of sulphoaluminate cement-based materials for rapid construction in low temperature environments, but also provide a theoretical basis for low temperature engineering applications.

## 2. Materials and Methods

### 2.1. C_4_A_3_S Synthesis

Calcium sulfoaluminate was synthesized by the following protocols: Chemical reagents, CaCO_3_, Al(OH)_3_ and CaSO_4_·2H_2_O, were dry-mixed in a ball mill for 30 min at a stoichiometric proportion of 3:6:1. Then, pastes were prepared by mixing the blends with 5% distilled water, which were subsequently pressed into cylindrical molds with a size of φ50 mm × 10 mm. These cylinders were dried in an oven at 105 °C for 12 h and then demolded. Subsequently, the dried cylinders were placed in corundum crucibles and sintered at 1320 °C for 1.5 h in a high temperature electric furnace to obtain clinkers, which were cooled immediately to ambient temperature by air flow. Finally, the clinkers were ground to pass an 80 μm mesh sieve and reserved in a sealed container.

### 2.2. Antifreeze Preparation

This work has been in a low temperature environment since the raw material is stirred. In order to ensure the water activity at low temperatures and the consistency of various performance test conditions, antifreeze was configured. First, we adjusted the concentration of sodium nitrite solution at −15 °C and different flow states of sodium nitrite solution after 24 h, then we selected the content of sodium nitrite as 10%. On the one hand, the hydration reaction can be maintained continuously; and on the other hand, it can prevent water freezing from affecting the cement strength.

### 2.3. Compressive Strength Test

Sulphoaluminate cement was made by mixing C_4_A_3_S and CSH_2_ in a molar ratio of 1:2, then mixing with 40% antifreeze, and pouring into the mixer. When mixing, it was first stirred at a low speed for 60 s, kept for 30 s, and then stirred at high speed for 60 s. Finally, the slurry was formed in a mold with a size of 20 mm × 20 mm × 20 mm, wrapped in plastic wrap, and cured in the corresponding temperature environment.

The mold was removed carefully after 1 day to ensure that the shape of each sample was complete without obvious cracks. The sample was then wrapped with plastic wrap and put in the corresponding temperature environment for curing to 3 days, 7 days and 28 days of age. Each set of strength test selected 6 samples to take the average value to reduce errors.

The model used in this work was a NJ-1068 cement paste mixer (Cangzhou Luyi Test Instrument Co., Ltd., Cangzhou, China). The technical parameters are: the low speed of the revolution of the mixing blade of 62 ± 5 r/min and high speed of 125 ± 10 r/min; and the low speed of the mixing blade’s autobiography 140 ± 5 r/min and high speed of 285 ± 10 r/min.

#### Sample Preparation

In order to accurately reflect the hydration characteristics of sulphoaluminate cement in low temperature environments, the raw materials, antifreeze and molds used in the test were placed in the corresponding temperature environments for 24 h in advance. It is worth noting that the tests at −15 °C and 0 °C always keep stirring-into-molding-forming in low temperature environments. The antifreeze was mixed into sulphoaluminate cement to maintain hydration.

### 2.4. Methods

#### 2.4.1. X-ray Diffraction

Bruker D8 Advance X-ray Powder Diffractometer (XRD, Bruker, Karlsruhe, Germany) with Cu-kα radiation (λ = 0.154 nm) over the range of 5 and 80° 2θ, with a step size of 0.02° and a step time of 0.5 s. For each sample, 10% α-Al_2_O_3_ was incorporated as an internal phase, and the obtained XRD patterns were subsequently analyzed by Rietveld method.

#### 2.4.2. Thermogravimetric Analysis

A TG/DTG thermogravimetric analyzer (NETZSCH STA 490C, Netzsch, Selb, German) was used to determine the mass loss as a function of the temperature. A small amount of ground powder, around 20 mg, was placed in corundum crucibles for analysis. N_2_ atmosphere was used as the carrier gas, with a flow rate of 100 mL/min. The experiment was performed over the range of 40 °C to 500 °C at a heating rate of 10 °C/min. Each batch was conducted three times for acceptable repeatability.

#### 2.4.3. Scanning Electron Microscopy

After the compressive strength tests, small sample pieces were collected and soaked in ethanol for 7 days, and dried at 40 °C for another 48 h. After that, the small sample pieces were sputtered with gold-coating before SEM (Quanta FEG 650) (American FEI Company, Hillsboro, OR, USA) observation.

#### 2.4.4. Mercury Intrusion Analysis

Mercury intrusion porosimeter was used to determine the porosity characteristics of CSA cement pastes. We took a small piece of pure pulp that had undergone the hydration treatment and dried the sample in a vacuum dryer at 40 °C for 24 h to remove water from the pore structure. The sample was cut into cubes of about 10 mm to test the hole structure by mercury intrusion. The porosity and pore size distribution were measured using an AUTOPORE IV 9500 mercury indenter produced by Micromeritics Instrument Corp. (Noclos, SC, USA). The measurable range of pore diameter was 3 nm–400 μm.

## 3. Results

### 3.1. Phase Identification for the Clinker

**Figure 1:**
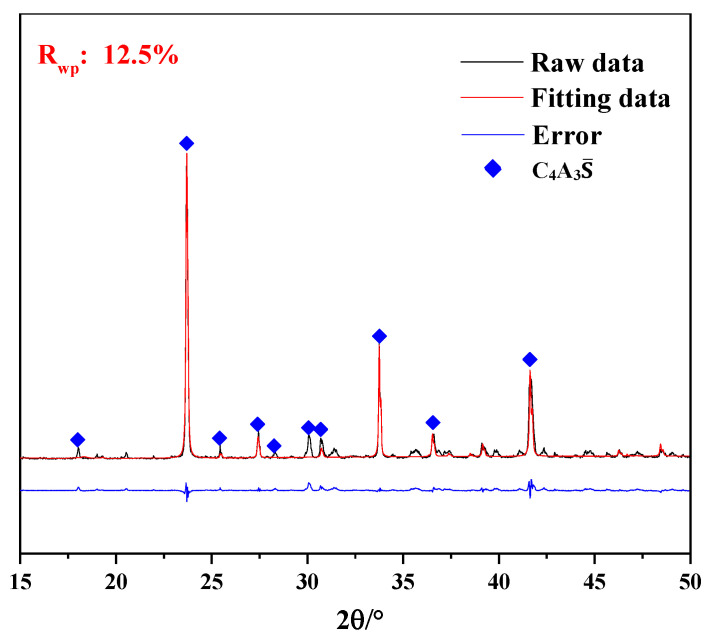
XRD patterns of C_4_A_3_S.

XRD qualitative information about the crystalline phases for the synthesized calcium sulfoaluminate clinkers was implemented by Rietveld method using TOPAS software. The XRD quantitative analysis results (Figure 1) showed that the main crystal phase of clinker was C_4_A_3_S with a purity of 96.02. The rest of the crystal phases were C_12_A_7_, CaSO_4_ and CaO, and their contents were 2.80%, 0.32% and 0.86%, respectively, which were not marked in the figure.

### 3.2. Hydration Products

**Figure 2:**
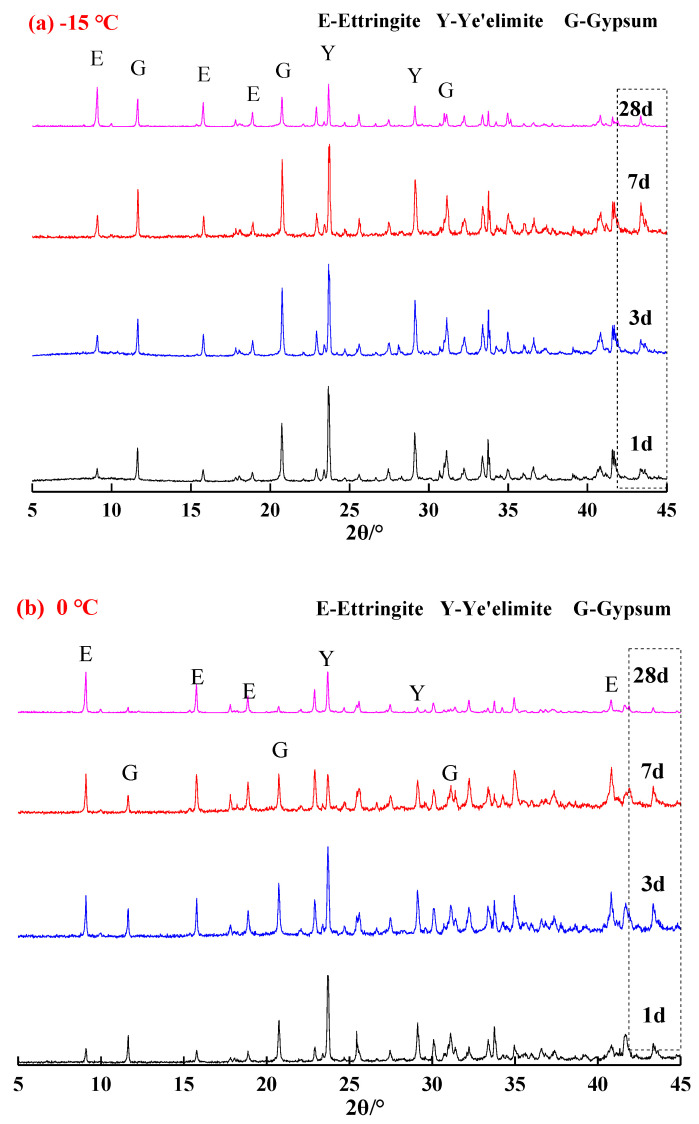

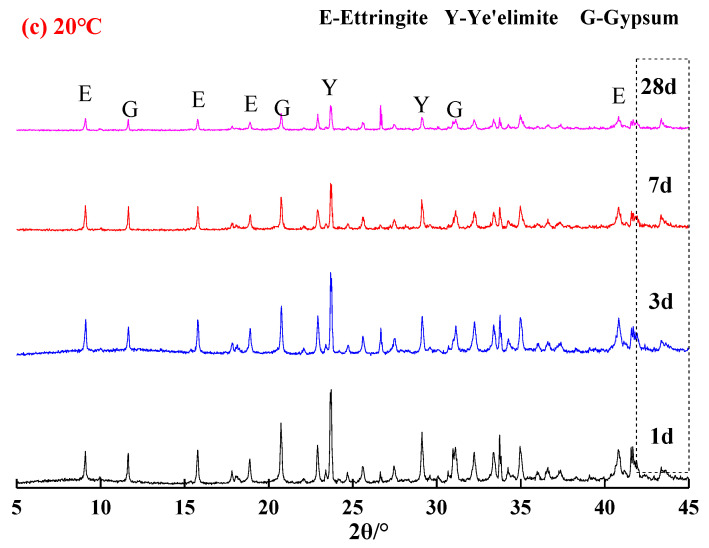
XRD patterns of samples at different temperatures: (**a**) −15 °C; (**b**) 0 °C; and (**c**) 20 °C.

Figure 2 presents the XRD patterns of the samples hydrated at corresponding temperatures of −15 °C, 0 °C and 20 °C to specified ages. It was confirmed that hydration phases of the C_4_A_3_S-CSH_2_ cement systems were ettringite, unhydrated anhydrous calcium sulfoaluminate and hydrated gypsum, which indicated that low temperatures were unable to change phases of hydration products and hydration carried out even at low temperatures of −15 °C and 0 °C. It should be noted that AH_3_ cannot be confirmed by the XRD patterns due to its amorphous state. However, a broad but weak characteristic peak could be observed in the range of 20°–21° 2*θ*, which very likely corresponded to the formation of AH_3_ phase [13]. XRD is thus not viable for identifying the existence and quantity of AH_3_, TG-DTG is more suitable and will be discussed at the end of this section.

In comparison with the samples hydrated at 20 °C and −15 °C (Figure 2a,c), higher amounts of ettringite were observed in the sample that was hydrated at 0 °C (Figure 2b), especially after 28 days curing. It is consistent with the Rietveld quantitative analysis and TG/DTG results (Table 1), for instance, the ettringite content at 28 d was 20.0%, 54.0% and 36.6% for the samples hydrated at −15 °C, 0 °C and 20 °C, respectively. For the samples hydrated at 20 °C for one day of curing (Figure 2c), the hydration reacted rapidly and a quite strong characteristic peak for ettringite was identified. In contrast, the characteristic peaks for ettringite in the samples that were hydrated at −15 °C and 0 °C (Figure 2a,c) were not that evident. It implied that the low temperature delayed the hydration of the C_4_A_3_S-CSH_2_ cement system at an early stage. Additionally, stronger characteristic peaks of unreacted ye’elimite and gypsum (Figure 2a) were observed in the samples hydrated for 3 d at −15 °C, compared to the samples hydrated at 0 °C and 20 °C at the corresponding curing ages. It was very likely attributed to the decrease in the dissolution rate of the sulfate by the low temperature, and therefore the formation of ettringite was delayed.

In particular, with regard to the samples hydrated at 0 °C, the characteristic peaks for ye’elimite decreased gradually, however, the characteristic peaks for ettringite increased with the curing age. This demonstrates that hydration of the C_4_A_3_S-CSH_2_ cement system at 0 °C continues to carry out at a high rate. At the stage of 28 days, gypsum was almost unidentified, which was consistent with the decrease trend of ye’elimite. It was concluded that the C_4_A_3_S-CSH_2_ cement system hydrated at 0 °C favored the formation of ettringite.

**Figure 3:**
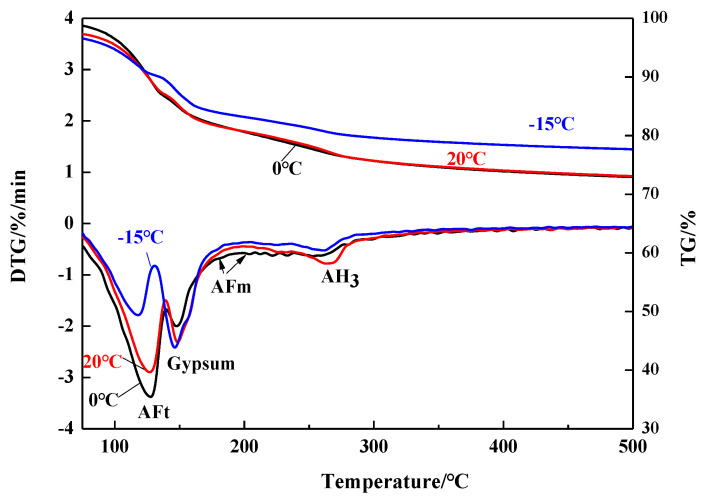
TG/DTG curves of samples hydrated for 28 days at different temperatures.

To evaluate the quantity of AH_3_ as stated above, TG/DTG method was applied and Figure 3 demonstrates the TG/DTG results of the C_4_A_3_S-CSH_2_ cement systems at 28 days. The TG/DTG results curves showed three evident mass loss peaks, which correspond to ettringite, gypsum and AH_3_, respectively, as indicated in Figure 3. Traditionally, ettringite decomposes over the range of 100–150 °C and gypsum dehydrates at around 150 °C [1,13,24]. Accordingly, the dehydration of the AH_3_ was carried out around 250–280 °C [1,25]. In addition, weak mass loss peak was observed at around 160–200 °C. The weak mass loss peak was assigned to AFm, as confirmed by literature [26].

AH_3_, especially, tends to transform from amorphous phase to regular crystal as gibbsite forms over 40 °C, as discussed in previous studies [1,27]. The water content during the transformation is, however, assumed to be constant [1], where three moles of water is included in one mole of AH_3_ phase. Therefore, the AH_3_ content can be calculated by Equation (1):

*ω_A_* = *kω_H_* (1)

where *ω_A_* is the content of AH_3_, and *ω_H_* is the bound water of AH_3_ obtained by TGA. Besides, *k* = *M_A_*/3*M_H_*, where *M_A_* is the molar mass of AH_3_ in g/mol, and *M_H_* is the molar mass of H_2_O in g/mol.

**Figure 4:**
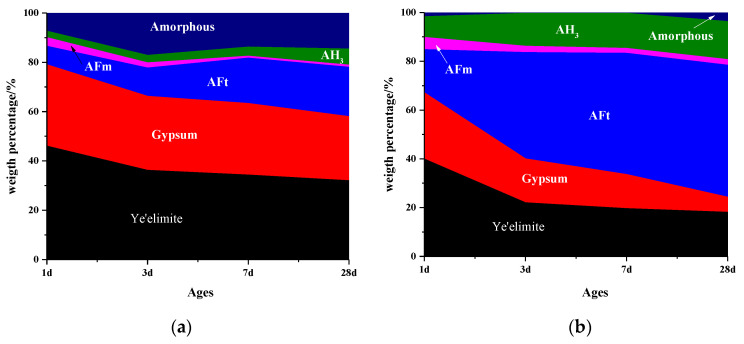

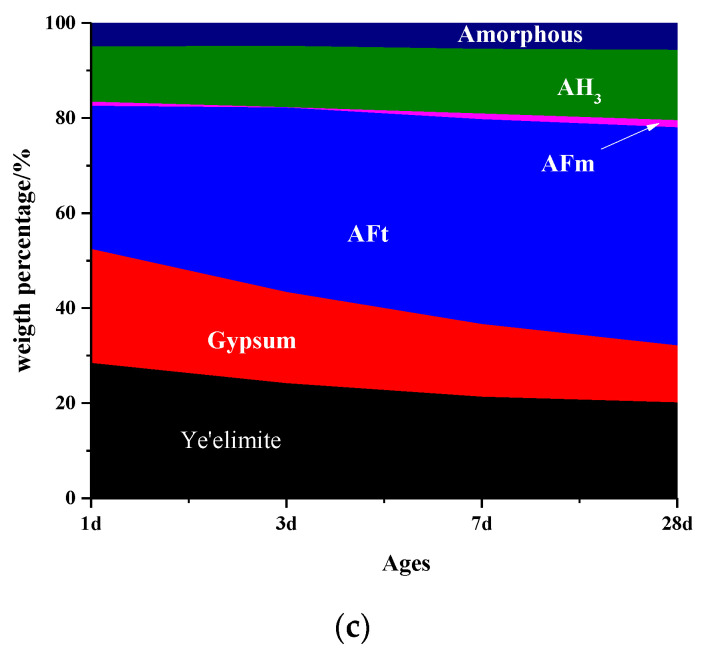
Phase composition of samples at different temperatures: (**a**) −15 °C; (**b**) 0 °C; and (**c**) 20 °C.

Figure 4 shows the mass percentage of C_4_A_3_S-CSH_2_ cement system determined by Rietveld method coupled with TG/DTG analysis. It is consistent with the mass loss tendency of AFt, gypsum and AH_3_ calculated by TG/DTG. It can be seen from Figure 4 that more AFm was formed at a very early stage for the sample hydrated at −15 °C and 0 °C, compared to the sample that was hydrated at 20 °C. As the ages increased, the AFm was gradually consumed. In addition, AFt were observed for all the samples at an early stage, even for that at −15 °C. It proved again that the hydration of C_4_A_3_S-CSH_2_ cement systems could be carried out at as low temperatures as at −15 °C. Moreover, the content of AFt for samples at −15 °C and 0 °C were lower than that at 20 °C at one day. After that, the AFt at low temperatures increased rapidly, especially for the sample at 0 °C in which AFt at 28 days was higher than that at 20 °C. It was confirmed that the low temperature of 0 °C was more favorable for the formation of AFt.

### 3.3. Hydration Degree Analysis

**Figure 5:**
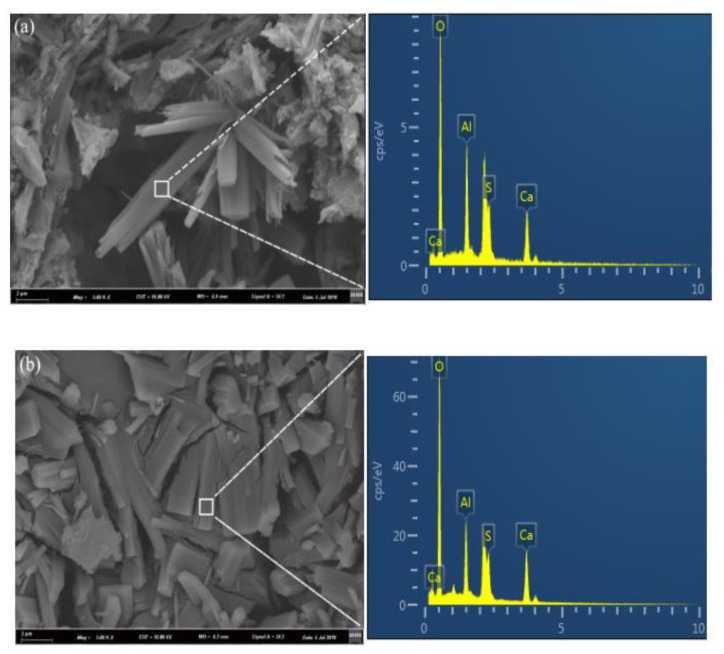

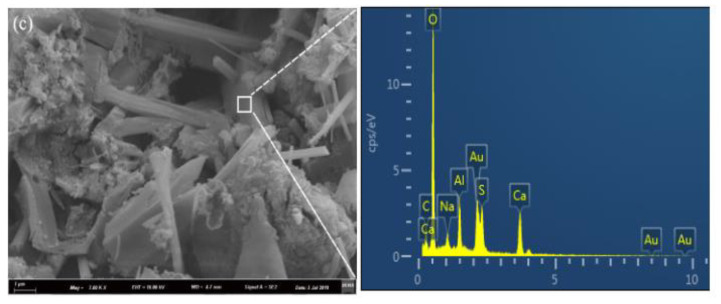
SEM images of samples hydrated for 28 days: (**a**) −15 °C; (**b**) 0 °C; and (**c**) 20 °C.

The hydration degrees of C_4_A_3_S hydrated at −15 °C, 0 °C and 20 °C were evaluated according to Equation (2) and are presented in Figure 5.

(2) $$D=1-\frac{w_{C_{4}A_{3}\bar{S}}}{w_{w_{C_{4}A_{3}\bar{S}}}^{0}}$$

where *D* denotes the hydration degree of C_4_A_3_S, %. $W_{C_{4}A_{3}\bar{S}}^{O}$ and $W_{C_{4}A_{3}\bar{S}}$ are the weight percentages before and after hydration, respectively.

Table 1 presents the hydration degrees of C_4_A_3_S cured at −15 °C, 0 °C and 20°C at different hydration stages. It was confirmed that the hydration degrees of the samples at −15 °C and 0 °C for one day of curing were 17.5% and 39.6%, respectively, which are significantly lower than the samples hydrated at 20 °C (Equation (3)). However, the hydration degrees of the samples at −15 °C and 0 °C beyond one day develops quickly, especially at 0 °C where the hydration degree at 28 days was higher than that at 20 °C. Nevertheless, the AFt at −15 °C for 28 days of curing was still far lower than at 20 °C. It demonstrates that the low temperature of 0 °C is more favorable for the hydration of C_4_A_3_S. It was in well agreement with phase quantitative results of the AFm and AFt formation of C_4_A_3_S-CSH_2_ cement systems determined by XRD and TG/DTG analysis in Figure 4. It shows that AFm content of samples at low temperatures are higher than that at 20 °C at early ages. Meanwhile, the AFt content of the sample at 0 °C exceeded that at 20 °C at later stages, however, the AFt at −15 °C was far lower than that at 20 °C. A plausible explanation is that the dissolution of sulfate decreased at the very early stage due to the low temperature. Meanwhile, C_4_A_3_S reacts right after contact with water to form AFm and AH_3_ (Equation (4)). Then, subsequent formation of SO_4_^−^ ions by dissolution of CSH_2_ react with AFm to form AFt. (Equation (5)).

C_4_A_3_S + 2CSH_2_ + 34H → C_6_AS_3_H_32_ (AFt) + 2AH_3_ (3)

C_4_A_3_S + 18H → C_4_ASH_12_ (AFm) + 2AH_3_ (4)

AFm + 2CSH_2_ + 16H → AFt (5)

Depletion of AFm further promotes the hydration of C_4_A_3_S. However, lower temperatures, such as hydration at −15 °C, can greatly reduce the overall dissolution of C_4_A_3_S, which inversely slows down the formation of AFm to some extent. It explains why the content of AFt for the sample at 0 °C is higher than that at 20 °C at the later stage, while the AFt at −15 °C was far lower than at 20 °C.

### 3.4. Scanning Electron Microscopy Analysis

The microstructures coupled with EDS results for the C_4_A_3_S-CSH_2_ cement system hydrated at −15 °C, 0 °C and 20 °C at the age of 28 days were presented in Figure 5. The EDS microanalysis results show that the area of the interests for the samples at −15 °C, 0 °C and 20 °C share the common elements of Ca, O, Al and S, and the ratios of Ca, O, Al and S are close to the theoretical ratio for ettringite. It implied that the crystals with different morphologies could be the ettringite. In addition, the SEM microphotographs show that the temperature can greatly affect the morphologies of the resulting ettringite crystals. For the samples hydrated at −15 °C, loose microstructures with needle-like ettringite crystals were observed. In contrast, the ettringite crystals in the sample at 0 °C was columnar and formed a compacted structure; it was consistent with the compressive strength development which will be discussed in Section 3.5.

Additionally, a loose microstructure with needle like crystals and amorphous phases was observed in the sample at 20 °C. Taking the hydration degree and compressive strength (as will be discussed in Section 3.5) results into account, the hydration degree at 28 days for the sample at 20 °C had a value of 66.8%, while the compressive strength was not as high as the sample at 0 °C. Previous studies [22,23] reported that low temperatures delayed the hydration of the cement which lowered the huge and concentrated heat release. Moreover, a reduction in the temperature differences between the external and internal samples favors the formation of well-developed AFt crystals, and it further contributes to the compressive strength. In contrast, high temperature undesirably enhances the hydration rate of the system, where hydration products rapidly formed and volume expansion, thus, likely occurs due to the formation of ill-timed crystals.

### 3.5. Compressive Strength Analysis

**Figure 6:**
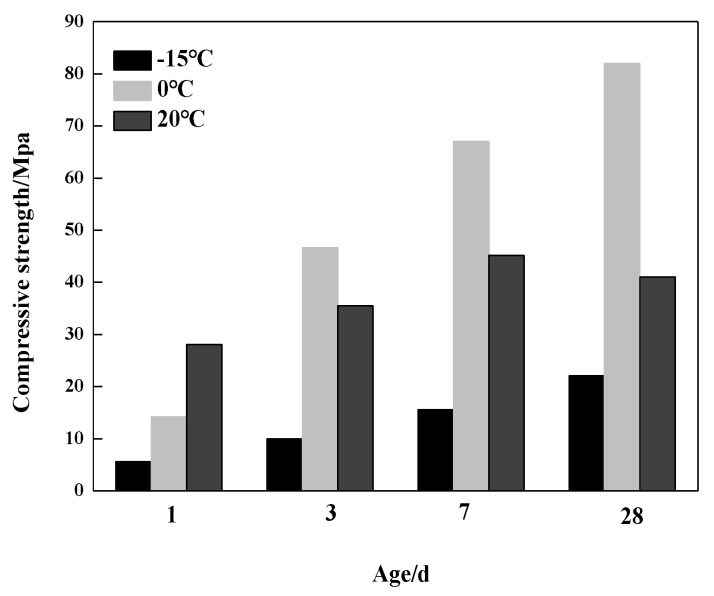
Compressive strength development of the samples at different temperatures.

Figure 6 shows the compressive strength development of the samples hydrated at −15 °C, 0 °C and 20 °C. It can be seen that the compressive strength of the samples hydrated at −15 °C and 0 °C for one day were 5.6 MPa and 14.2 Mpa, respectively, which were merely about 20% and 50% of the sample hydrated at 20 °C. It implied that lower temperature reduced the hydration rate of the C_4_A_3_S-CSH_2_ cement system at the very early stage of one day, leading to lower compressive strength. Nevertheless, the compressive strength of the sample hydrated at 0 °C increased rapidly beyond one day. It demonstrated that a 0 °C hydration environment was more favorable for the compressive strength development of the C_4_A_3_S-CSH_2_ cement system. The results are in well agreement with the hydration degree of the C_4_A_3_S, determined by XRD analysis of the hydration degree increments and contents of AFt at 0 °C.

As the hydration ages increased, the compressive strength of the samples hydrated at 0 °C for 28 days reached the maximum, which was significantly higher than that at 20 °C. In contrast, the compressive strength of the samples hydrated at −15 °C was only 22.1 MPa, and it was comparably lower than the two other temperatures. Of particular note, a significant reduction in the compressive strength of the sample hydrated at 20 °C for 28 days was observed, compared to that at seven days. It indicates that adding gypsum into the C_4_A_3_S-CSH_2_ cement system at ambient temperature will lead to the decrease in its compressive strength. It was very likely due to the micro expansion of the sample at the later hydration stage.

To examine the effect of hydration products of the C_4_A_3_S-CSH_2_ cement system on compressive strength, Table 2 shows the compressive strength and AFt content of the C_4_A_3_S-CSH_2_ cement system at −15 °C, 0 °C and 20 °C. It can be seen that the low temperature can greatly decrease the AFt content at one day, compared to that at 20 °C, leading to lower compressive strength. At three days, the hydration of the C_4_A_3_S-CSH_2_ cement system at 0 °C continued and was carried out at a high rate. AFt increased, corresponding to a significant enhancement on the compressive strength. There was a relatively weak correlation between the formation of AFt and compressive strength, in terms of the C_4_A_3_S-CSH_2_ cement system hydrated at 20 °C for 28 days.

**Figure 7:**
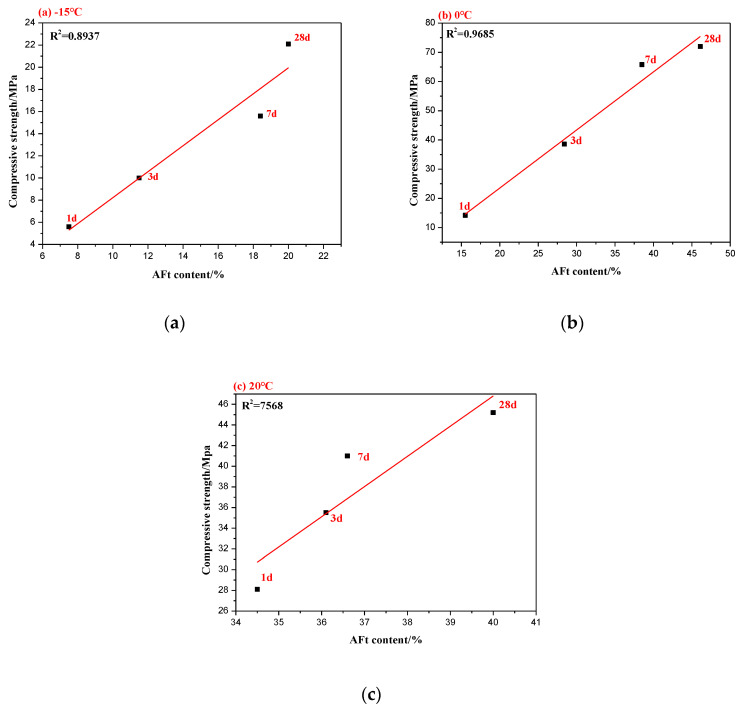
Relationship between the AFt content and compressive strength: (**a**) −15 °C, (**b**) 0 °C; and (**c**) 20 °C.

Figure 7 displays the relationship between the content of AFt and compressive strength at the corresponding ages. R^2^ is the goodness of fitting, and R^2^ closer to 1 indicates better fitting and stronger correlation. It was concluded that there was a positive correlation between the compressive strength at an early stage and the formation of AFt for the C_4_A_3_S-CSH_2_ cement system hydrated at low temperatures. Specifically, the compressive strength increased with the formation of AFt. At low temperatures, the hydration products of the C_4_A_3_S-CSH_2_ cement system at early stage were ettringite (AFt) and aluminate gel (AH_3_), however, the AH_3_ in early structures had difficulty providing strength [14,28]. Ettringite forms a rigid framework structure, and the mechanical bond and interlocking bond between ettringite crystals play an important role in the strength. However, at later stage for hydration at low temperatures, the correlation between the compressive strength and AFt content becomes weak.

Additionally, it can conclude that the compressive strength tends to focus on the microstructures of the matrix. The compressive strength of the C_4_A_3_S-CSH_2_ at −15 °C was as low as 22.1 Mpa, and correspondingly, its hydration product AFt develops into acicular crystals, which makes the microstructure loose. The compressive strength of the C_4_A_3_S-CSH_2_ cement system at 0 °C reached its maximum value (82 MPa), and its hydration products (Aft) were columnar crystals which formed a denser microstructure. It can be seen that the microstructures play a more important role on the compressive strength at later stages. The compressive strength development was determined by many factors. In addition to the amount of AFt and the microstructure of the hydration products, there are also factors such as porosity, which is related to the strength of calcium sulfoaluminate cement.

### 3.6. Mercury Intrusion Analysis

**Figure 8:**
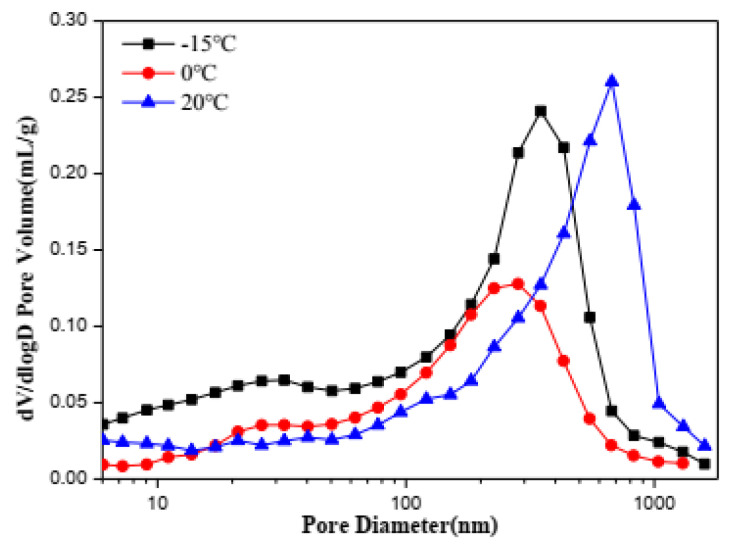
Pore size distribution of samples hydrated for 28 days.

Finally, mercury intrusion porosimeter was used to further prove the correlation between strength and pore structure. The pore size distribution (PSD) of C_4_A_3_S-CSH_2_ cement pastes cured for 28 d at −15 °C, 0 °C and 20 °C investigated in this work are plotted in Figure 8. From this, it can be seen that the C_4_A_3_S-CSH_2_ cement pastes hydrated for 28 days at −15 °C, 0 °C and 20 °C had mode diameter values of 349 nm, 283 nm and 675 nm, respectively. Compared with the C_4_A_3_S-CSH_2_ cement pastes hydrated for 28 days at room curing, the mode pore size was reduced for low temperature environments. The low-temperature environment at 0 °C was more inclined to obtain the lowest mode diameter.

**Figure 9:**
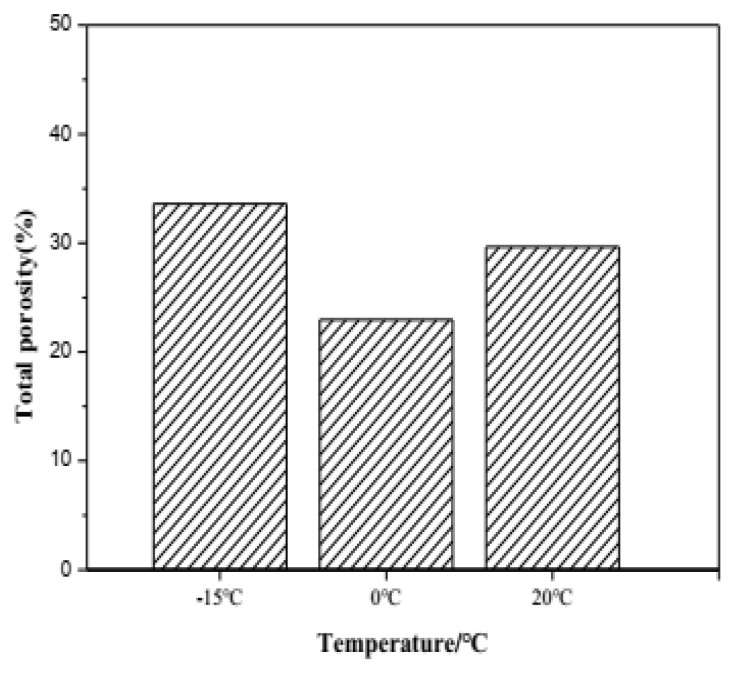
Porosity characteristics of samples hydrated for 28 days at different temperatures.

The total porosity of samples hydrated for 28 days, cured at −15 °C, 0 °C and 20 °C, determined by mercury intrusion analysis (MIP) is illustrated in Figure 9. It can be seen that the low curing temperature demonstrates a significant effect on the total porosity of C_4_A_3_S-CSH_2_ cement pastes. The lowest porosity happens in cement pastes cured at 0 °C, while the highest porosity is shown in cement pastes cured at 20 °C. The result is well consistent with the similar literature study [28], that a low temperature is in favor of denser and more homogeneous matrices due to continuous substantial hydration. Under the low temperature (−15 °C), the hydration reaction of C_4_A_3_S-CSH_2_ cement is greatly inhibited, resulting in a decrease in the ettringite content of the hydration products, and small amount of product fills the internal structure, which makes the porosity 33.6% belowe 0 °C. This work further proved the high strength at 0 °C.

## 4. Conclusions

This work systematically investigated the effect of low temperature on the hydration properties of C_4_A_3_S-CSH_2_ cement systems by the XRD, TG-DSC, SEM, MIP and Rietveld quantitative analysis methods. The following conclusions are drawn:

- Hydration of the C_4_A_3_S-CSH_2_ cement system could be carried out at low temperatures, even at temperatures as low as −15 °C. The main hydration product at −15 °C, 0 °C and 20 °C was ettringite, together with a small amount of AFm and AH_3_. Low temperatures were unable to change the types of the hydration products, but the low temperature of 0 °C was more favorable for the formation of ettringite.
- At low temperatures of −15 °C and 0 °C, the early hydration of the C_4_A_3_S-CSH_2_ system could be inhibited. However, hydration of the system at 0 °C continued beyond one day and the hydration degree was higher than that at 20 °C.
- The morphologies of the AFt for the C_4_A_3_S-CSH_2_ cement system could be altered by different low temperatures. The AFt develops as needle-like structure at −15 °C to form a loose structure, while it develops a columnar structure at 0 °C to form a denser structure.
- A low temperature was not conducive to the compressive strength of the system at a very early stage, but the compressive strength developed rapidly at the later stage for the system at 0 °C. For hydration at 28 days, the compressive strength at 0 °C reached its maximum, which was significantly higher than that at 20 °C. There was a positive correlation between the early compressive strength and AFt content. The higher the early strength of the cement system, the greater the ettringite content. The later compressive strength tended to depend on the microstructure development and porosity.

## Figures and Tables

**Table 1:** Calculated hydration degree of C_4_A_3_S cured at different temperatures.

Age	−15 °C	0 °C	20 °C
1 day	17.5%	39.6%	53.1%
3 days	33.3%	63.9%	60.3%
7 days	36.8%	68.8%	63.6%
28 days	40.1%	72.2%	66.8%

**Table 2:** Compressive strength and AFt content of the C_4_A_3_S-CSH_2_ cement system.

Sample	1 Day		3 Days		7 Days		28 Days	
	Compressive Strength/MPa	AFt/%	Compressive Strength/MPa	AFt/%	Compressive Strength/MPa	AFt/%	Compressive Strength/MPa	AFt/%
−15 °C	5.6	7.5	10.0	11.5	15.6	18.4	22.1	20.0
0 °C	14.2	23.2	46.6	39.5	67.0	43.6	82.0	54.0
20 °C	28.1	34.5	35.5	36.1	45.2	40.0	41.0	36.6
